# Optimizing Emergency Department Operations: A Simulation Framework for Managerial Decision-Making

**DOI:** 10.1155/jonm/8874680

**Published:** 2025-09-18

**Authors:** Eman Ouda, Andrei Sleptchenko, Mecit Can Emre Simsekler

**Affiliations:** Department of Management Science & Engineering, Khalifa University of Science & Technology, Abu Dhabi, UAE

**Keywords:** design thinking, digital twin, discrete event simulation, emergency department, experimental design, future of healthcare, healthcare

## Abstract

Emergency departments (EDs) are frequently challenged by fluctuating patient volumes and resource constraints, leading to overcrowding and operational inefficiencies. This study presents a framework that combines design thinking and discrete event simulation (DES) to optimize ED processes and improve patient outcomes. By using both methods in conjunction with the design of experiments, the digital twin evaluates the effects of various resource configurations on key performance indicators, such as length of stay (LOS). The model highlights critical resource bottlenecks and explores alternative scenarios to improve ED performance. A case study demonstrates that targeted adjustments in nurse staffing and bed management significantly improve operational productivity and reduce patient LOS. The proposed approach provides hospital administrators with practical information to optimize resource allocation and streamline ED operations, contributing to better patient experience. This methodology is particularly suited for healthcare systems that face different patient demands and limited resources.

## 1. Introduction

Overcrowding in emergency departments (EDs) remains a persistent challenge, negatively impacting quality of care, patient satisfaction, and the work environment of healthcare providers. This ongoing issue has sparked growing interest in leveraging experimental design methodologies to develop innovative solutions. According to Sun et al. [1], high levels of ED crowding are associated with increased inpatient mortality, longer length of stay (LOS), and higher costs for admitted patients. Addressing similar challenges, the UAE's healthcare system has implemented regulations aimed at reducing ED overcrowding [2].

This study represents the first effort in the UAE to analyze patient flow and its regulatory framework using an integrated design thinking (DT) approach, combined with design of experiments (DOEs) and discrete event simulation (DES). The aim is to develop and apply a methodology-driven framework that addresses real-world problems in ED operations, supports decision-making, and evaluates the effects of proposed changes in a virtual environment before implementation.

Emphasizing a human-centered approach to problem-solving, DT has shown considerable potential in various domains, including healthcare [3]. As highlighted by Ortíz-Barrios and Alfaro-Saíz [4], strategies such as computer simulation and lean manufacturing have proven effective in addressing the major operational challenges in EDs. Similarly, DOE has been recognized as a powerful tool for improving product quality by identifying significant factors affecting product lifetime and selecting optimal factor levels for the analysis and managerial highlights [5]. For more than 5 decades, researchers have applied simulation modeling tools to address overcrowding, although specific goals and methods have varied widely between studies [6–8]. Despite this, a unified approach that effectively combines these methodologies remains underdeveloped.

The primary contribution of this paper is a digital twin-based DT framework that integrates regression modeling and DES. Unlike previous studies by [9] and Baril et al. [10], which have explored isolated methodologies, this research takes a more holistic approach by incorporating DES alongside DT to optimize ED processes. This integrated methodology allows testing and process designs, with a strong emphasis on human-centered solutions and interdisciplinary collaboration. The paper demonstrates the successful implementation of this approach, in which healthcare providers and managers improve patient outcomes.

By offering a comprehensive methodology that merges DT, DOE, and DES, this paper contributes to ongoing efforts to resolve ED overcrowding. The key contributions of this research include the following:

• Developed a digital twin-based DT framework incorporating DOE and DES to improve ED operations (see Table 1).
• A focus on human-centered approaches to systematically test and optimize process designs.
• The potential for interdisciplinary collaboration to enhance patient outcomes.
• Practical implications for healthcare providers and managers through this interdisciplinary approach.

The remainder of this paper is organized as follows.  Section 2 provides an overview of DT, DOE, and DES, along with their prior applications.  Section 3 introduces the integrated DT framework, which addresses key challenges related to resource management and improvement of hospital outcomes. In Section 4, a case study is presented to demonstrate the effectiveness of the framework.  Section 5 outlines the mathematical results and proposed solutions. Finally, Section 6 summarizes the findings, discusses theoretical and practical implications, and highlights limitations as well as potential avenues for future research.

## 2. Background and Literature Review

### 2.1. Overview of DT

According to DT, innovation projects should prioritize human needs, focusing on the emotions, fears, and desires of users [11]. By placing people at the center of the design process, this approach reduces the risk of failure and improves the likelihood of successfully implementing proposed solutions [12]. In healthcare, Roberts et al. [13] demonstrated that DT facilitates the development of new approaches to complex and persistent challenges through human-centered research, diverse teamwork, and rapid prototyping.

As a co-creation model, DT engages all stakeholders in the design process, making it particularly well-suited for improving ED processes. By focusing on understanding the needs of patients, staff, and other stakeholders, it generates a range of ideas, prototypes, and iterative testing, which are refined based on feedback. Additionally, studies recommend nurse managers to address organizational factors and support similar frameworks for better care outcomes [14]. The importance of designing healthcare processes from the patient's perspective has been highlighted as a key factor in driving meaningful improvements [15, 16].

For example, Dosi et al. [9] explored the application of a DES model with a DT process in a major ED in northern Italy. The goal was to improve key performance indicators (KPIs) and improve the quality of work of healthcare professionals. Within 18 months, this combined approach resulted in a project supported by the ED, illustrating DT and simulation tools in healthcare.

Successful and lasting innovations require participatory design inquiries that challenge the underlying assumptions and norms in service delivery. This means engaging the right stakeholders at critical moments and empowering them to co-create flexible platforms that they can own and adapt over time [16]. These paradigmatic changes are becoming increasingly important in public service reforms, particularly in healthcare, where design practice and research play a crucial role in this shift. Although DT was originally introduced as a cultural approach for organizations, early studies focused on specific tools and methods to solve management challenges [17], further highlighting its versatility and value in various organizational contexts.

### 2.2. Overview of DOE

A systematic methodology used to explore and test variables to determine the optimal configuration to maximize desired results is DOE. For example, Dessouky and Bayer [18] integrated DOE into building design to minimize maintenance costs, using a 2-factorial design to identify key factors affecting costs and applying the Taguchi loss function to improve the maintainability of buildings. Similarly, in a healthcare context, Visintin et al. [19] used DOE and simulation in a pediatric ED, showing that anticipated treatment, where triage nurses prescribed tests for nonurgent patients, reduced LOS by 44.6% for respiratory and 14.5% for trauma patients, outperforming traditional protocols. Additionally, DOE has been successfully applied to improve processes such as patient flow and staffing levels in EDs [20]. By systematically varying key factors, DOE helps identify the variables that most significantly influence ED performance.

Several studies have demonstrated the effectiveness of DOE in improving ED processes. For instance, Kolker [20] applied DES and DOE to reduce patient LOS in the ED, finding that optimizing LOS can significantly decrease patient wait times and minimize ambulance diversions, thus improving overall ED performance. Similarly, Atalan and Dönmez [21] used DES and DOE to optimize resource allocation in a case study, resulting in reduced patient waiting times and an increase in the number of treated patients. This approach provides a practical and cost-effective way to improve the management of healthcare resources.

Using DOE to test the impact of different factors on ED operations, healthcare managers can identify critical opportunities for improvement and develop optimized process designs. DOE, merged with DT and DES methodologies, provides a clear strategy for improving ED operations. For example, Wang et al. [22] analyzed patient flow as a closed-loop process with limited resources, using DOE to calculate LOS and explore system properties. These uses of DOE highlight its worth in optimizing processes in intricate healthcare settings, ultimately enhancing care quality and patient experience.

### 2.3. Overview of DES

Simulation has been widely used to improve EDs, and many studies have shown success in improving workflows. It serves as a powerful method for analyzing healthcare systems, both in small and large populations, providing valuable information to policymakers [23, 24]. Despite the growing number of studies on simulation models, more research is needed to fully assess their value in healthcare. For example, while Fone et al. [25] conducted a systematic review of healthcare simulation models, they were unable to definitively conclude their value due to limited evidence of real-world implementation, highlighting the need for additional investigation.

Among the various simulation approaches, DES has been widely applied in the healthcare industry to improve the quality of care of the ED. DES models are particularly suited to simulate complex systems, such as patient flow and resource allocation, making them highly valuable for identifying process bottlenecks and optimizing staffing levels [24, 26]. For example, studies by Ouda et al. [27] used DES to optimize staffing levels and reduce patient waiting times. Similarly, Taboada et al. [28] developed an agent-based simulator to serve as a decision support system for ED managers, focusing on optimizing staff resources, including triage nurses, admission staff, and physicians.

DES has also been used to evaluate the impact of specific interventions and disaster preparedness. For example, Gul and Guneri [8] conducted a comprehensive review of the literature on ED simulation applications, covering both normal operations and disaster scenarios. They used DES and DOE to assess the ED network's readiness for an earthquake and disaster response. In addition, DES has been used to assess the impact of targeted interventions on patient outcomes. Brenner et al. [29] developed a simulation model to identify bottlenecks in patient performance and determine the optimal number of staff and equipment resources required to improve results. In another study, Russ et al. [30] and Day et al. [31] used DES to explore the effects of adding a triage physician and an additional triage provider to reduce the LOS of the patient.

While there is considerable research on DES in healthcare settings, little has focused specifically on healthcare professionals, particularly in modeling nursing workload, care quality, and work environments [32]. Hospital managers often struggle to assess the impact of operational policies, highlighting the need for effective planning tools. Further research utilizing DES has provided additional recommendations for resource configuration. For instance, Oh et al. [33] developed a DES model to pinpoint bottlenecks in patient flow, leading to significant recommendations on resource configuration and operational policies. As a result, their interventions led to a substantial reduction in patient LOS, showcasing the practical value of DES.

### 2.4. Integration of Approaches

Simulation models have been used in various studies to improve ED processes. These models are used to predict patient flow and optimize resource allocation. To integrate DT, DES, and DOE, we built on a four-phase representation from Beckman and Barry [34] to illustrate the parallel use of DT and DES. This framework defines the DT process as a sequence of four phases: (1) comprehension, (2) abstraction, (3) ideation, and (4) testing and implementation. This sequence corresponds to the classical DES model [35], where the first phase involves structuring the problem; the second phase abstracts the model; the third phase experiments with different scenarios to identify the best-performing solution; and the fourth phase assumes that the optimal solution is acceptable to the organization.

Despite the numerous studies that address overcrowding in the ED and related issues, and the acceptance of simulation techniques and DT, there are still barriers to applying the results of these studies in practice [36]. This study builds upon the methodology proposed by Dosi et al. [9], who successfully implemented a combined DT and simulation approach in an ED. DES, DT, and DOE can significantly enhance ED processes by incorporating a user-centered design approach. The following sections will present the proposed methodology and its application in an ED setting.

## 3. Integrated DT Framework

**Figure 1:**
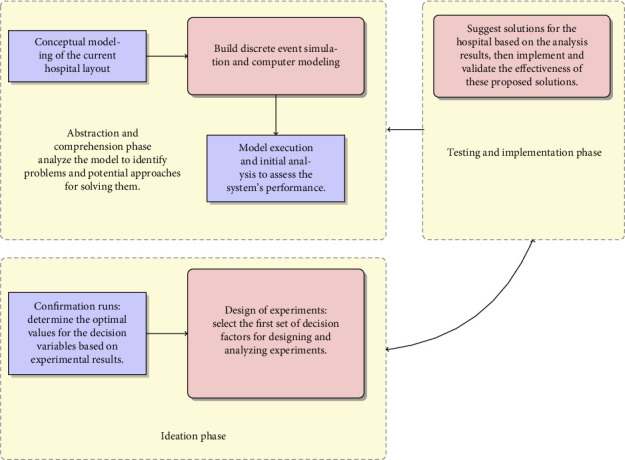
A visual representation of the methodology's framework, integrating conceptual modeling, discrete event simulation, design of experiments, and testing and implementation phase to optimize emergency department operations.

The proposed framework (Figure 1) operationalizes the concept of a digital twin by creating a virtual replica of the ED using DES. This digital representation is not connected in real-time but is informed by retrospective data, site visits, and expert input, allowing us to simulate and test various interventions in a risk-free environment. The digital twin framework combines DT with DES and DOE, progressing through multiple phases to identify, test, and implement solutions aimed at improving ED performance. The process begins by analyzing the existing hospital layout and developing a conceptual model specific for DES. Then, a detailed computer model of the ED was built, capturing patient flow, resource allocation, and staffing levels. Initial simulations were performed, running multiple replicas to confirm both patient and hospital performance measures under the baseline scenario. This provided the foundation for the ideation phase, where we selected decision factors for the DOE. Following the experiments, confirmation runs were performed using the optimal values of the decision variables identified through DOE, validating the proposed solutions. In the final testing and implementation phase, suggestions are given to hospital interventions based on these optimal values.

**Figure 2:**
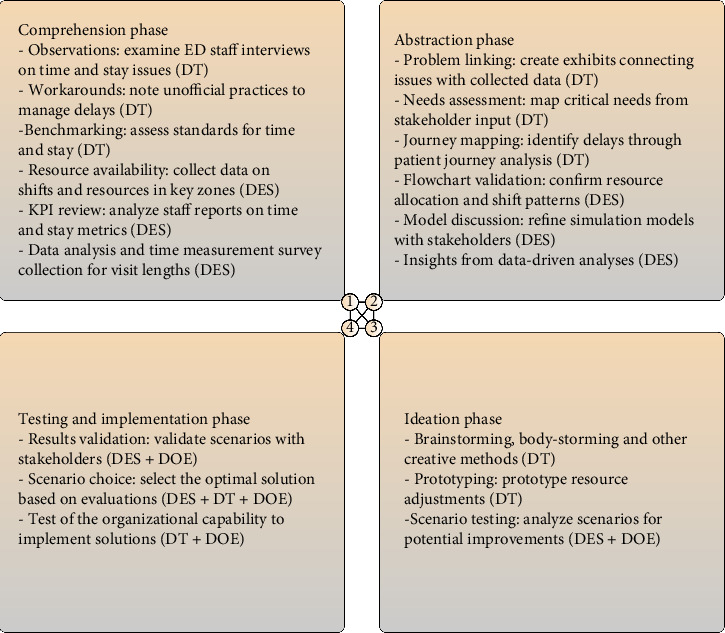
Activities performed in the emergency department for each phase: design thinking (DT), discrete event simulation (DES), and design of experiments (DOE).

The DT process, shown in Figure 2, served as a critical framework that guided each step. The process started with the comprehension phase, where data collection and team meetings were used to understand the structure and workflow of the ED. Qualitative data on patient pathways and the Emergency Severity Index (ESI) triage system, combined with quantitative timestamps, were incorporated into the DES model to ensure precision. During this phase, structured interviews with ED staff identified key issues such as excessive wait times and LOS, while benchmarking industry standards to identify performance gaps. Detailed data on staff shifts and resource availability were collected to further inform the model.

Next, the abstraction phase aimed to construct a high-level overview of the ED system and identify key challenges. A DES model was developed using the collected data to mirror real-world conditions. Exhibits and simulations were generated to correlate the observed problems with the data, identifying key areas for improvement, such as resource allocation inefficiencies and staffing issues. In the ideation phase, brainstorming sessions and prototyping were used to generate creative solutions. The DES model was used to simulate various scenarios, helping the team evaluate and refine the proposed changes. These sessions focused on optimizing nurse and bed resources, with DOE providing quantitative support for the recommended adjustments.

Finally, in the testing and implementation phase, the most promising scenarios were tested and validated through further simulations. DOE played a crucial role in selecting key factors that influence ED outcomes, ensuring that the recommended solutions were based on solid evidence. The simulation results were presented to the stakeholders, and the most effective solutions were selected based on a thorough evaluation of logistical, human, and resource considerations. This comprehensive approach ensured that the selected interventions were both practical and implementable, maximizing the likelihood of successful execution. In general, this integrated framework provides a structured and evidence-based methodology to improve ED operations. By combining DT, DES, and DOE, the approach allows thorough exploration, ideation, and validation of solutions, ultimately enhancing both operational efficiency and patient care in the ED.

## 4. Framework Application and Case Study

In this section, we outline the steps taken to generate and evaluate new process design options aimed at improving the performance of the ED of a regional hospital. Using the DT approach presented in Section 3, we sought to identify and address the root causes of the issues identified during the literature review and data analysis phases. The DT process involves iterative phases focused on understanding and solving problems, with the aim of identifying innovative solutions that can be practically implemented in a real-world ED setting.

### 4.1. Case Study: The ED of a Regional Hospital

To illustrate the proposed approach, we conducted a case study in the ED of a regional hospital. In this study, we focused on the adult and pediatric zones, excluding the resuscitation zone due to the unpredictability and difficulty in managing resources in that area. The hospital, located in the UAE, handled approximately 129,000 emergency cases and more than 27,000 inpatient admissions as of 2023 [37]. We analyze patient data from January to June 2022, using timestamps that mark key points in patient journeys, such as registration, triage, doctor consultations, bed assignments, and discharge, as shown in Table 2.

We employed both qualitative and quantitative analyses to gain understanding of the patient flow and resource utilization. The findings from these analyses informed the development of a simulation model aimed at improving ED operations.

**Figure 3:**
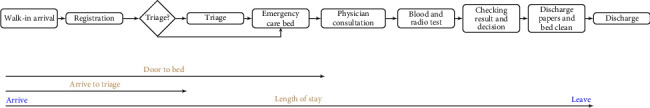
General patient flow model showing the resources required at each emergency care process.

The first two phases of DT, comprehension and abstraction, aim at developing a deep understanding of the problem space. During the comprehension phase, the team collected data through research and stakeholder interviews. A patient flow diagram (Figure 3) was developed, mapping each stage of the patient's journey from arrival to discharge. This diagram was informed by ethnographic research, including direct observation of ED processes and interviews with healthcare providers and support staff, with a focus on the patient experience and the identification of pain points in the system.

**Figure 4:**
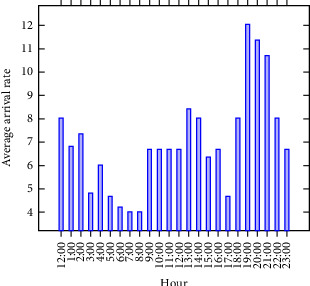
Average arrival rate for each case study.

In addition to qualitative insights, we conducted quantitative analyses on patient wait times and LOS. These analyses helped pinpoint bottlenecks in the ED process, further supported by a review of the literature on best practices in ED design and optimization. The average arrival rate of patients during the case study period is shown in Figure 4. Insights from the patient flow diagram and data analysis informed the creation of a DES model to systematically test and optimize process design options, including staffing levels and equipment availability.

In the abstraction phase, the team focused on identifying patterns and opportunities for improvement. The brainstorming sessions challenged existing assumptions, fostering creative thinking. Stakeholder mapping, particularly in the pediatric and adult zones, helped pinpoint crucial adjustments needed in staffing and resource allocation. For this study, we focused on ESI levels 3 and 4, which represent approximately 70% of the ED patient population. ESI is a widely adopted triage tool that categorizes patients based on the urgency of their medical condition, enabling efficient prioritization and resource use in EDs [38]. This phase laid the groundwork for the ideation phase, highlighting areas for targeted interventions.

### 4.2. Ideation Phase

The ideation phase is critical in the DT process, where ideas are generated and refined based on the comprehension and abstraction phases. In this study, the ideation phase focused on exploring how different resources, such as beds and staff, impact two key performance metrics: LOS and arrival-to-doctor time in both the adult and pediatric zones. To systematically explore these effects, using DOE, the team tested different process design options and evaluated their effects on the ED performance. Hence, DOE allowed us to test various combinations of resource availability and allocation, using the DES model to simulate the impact of these options. The exact combinations of these experimental conditions are detailed in Section 4.2.2 (Experimental Design). Each scenario was then simulated using our DES model to observe how different configurations impacted system performance. The simulation results, which illustrate the effect of each tested scenario, are presented in Section 5 (Results). This integration of DOE and DES enabled us to quantitatively assess and compare the impact of multiple design options and resource allocations before making data-driven recommendations for ED optimization.

#### 4.2.1. Simulation Model

The simulation process encompasses various components, including integrating predefined parameters subject to modification during runtime. These parameters consist of various factors such as schedule and availability and subsequently serve as the foundation for the simulation framework. Among these parameters are sample inputs, arrival times, and an evaluation of the number of available physicians, nurses, and other related personnel while accounting for the delay patients encounter. The simulation also involves a per-shift analysis, further reinforcing the importance of comprehensive parameterization and modeling techniques to yield the most accurate and valuable results.

**Figure 5:**
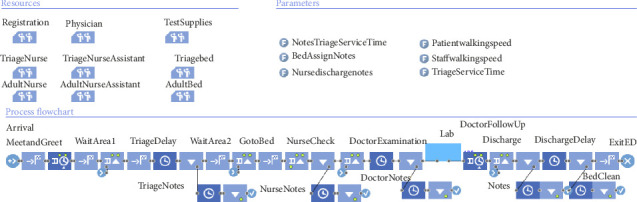
AnyLogic logic view of the emergency department simulation model, showing key resources, parameters, and process flow from patient arrival to discharge.

The DES model was built on the basis of the patient flow diagram and available data. The model incorporated detailed representations of patient journeys and resource usage, providing a visual simulation of the ED operations using AnyLogic software (Figure 5). The simulation model used a fixed random seed and ran up to 10 replications, each lasting approximately 3 min. Input service times are provided in Table 3.

To ensure accuracy, the model underwent thorough verification, validation, and calibration. Verification was conducted through direct collaboration with medical staff to confirm the correct implementation of logic and processes. Validation focused on assessing the model's ability to replicate actual patient flow in the ED by comparing simulated outputs with real-world data using the percentage difference formula (equation (1)). The simulation achieved a deviation of less than 5% (Table 4), indicating high precision:

(1) $$\begin{array}{c} \text{Percentage Difference}=\frac{\text{Simulated}-\text{Real}}{\text{Real}}*100. \end{array}$$

To enhance reliability, the model was executed on different dates over a 2-week period, allowing it to reach a steady state through multiple replications. Calibration was guided by timestamp data (Section 4.1), comparing the distributions of arrival-to-triage, arrival-to-doctor, and discharge times. Service time parameters were fine-tuned using the OptQuest optimization engine alongside real data. The calibration was validated through statistical techniques, such as distribution fitting, histograms, P-P plots, and Q-Q plots. While the results showed good overall agreement, minor discrepancies remained. These were likely due to real-world variability, simplifying assumptions in model design, and potential inconsistencies in input data.

#### 4.2.2. Experimental Design

The impact of key resources on LOS and arrival-to-doctor time was tested using DOE. LOS measures the total time a patient spends in the ED, from arrival to discharge, with a shorter duration indicating more effective care. Similarly, arrival-to-doctor time tracks how quickly patients are seen by a physician after arriving, with shorter times reflecting better service and timely care.

Two independent variables, the number of nurses and beds, were evaluated at three levels: normal allocation, an increase 25%, and a decrease 25% (Table 5). Nurses are vital for managing patient flow and performing tasks, such as triage, medication administration, and patient monitoring. Meanwhile, the number of beds determines the ED's capacity to accommodate patients. Specifically, the normal level represents the baseline levels of staffing and resources. Therefore, a 25% increase in resources is intended to improve efficiency and reduce patient wait times by increasing the availability of nurses and beds. In contrast, a 25% decrease is expected to prolong wait times and reduce operational efficiency within the ED. A full factorial design was implemented, resulting in experimental scenarios that systematically explored the combined effects of changes in staffing and bed capacity changes on performance results. This design enabled the identification of the main effects and the effects of the interaction between the two factors, providing information on the resource configuration that minimizes wait times.

## 5. Regression Analysis

This section presents four different regression analyses, each corresponding to one of the four dependent variables in this study, as detailed in Section 4.2.2.  Table 6 displays the regression results for all four analyses, highlighting the regression equations. Furthermore, the analysis of variance (ANOVA) for each regression model was examined, with the *F* values and the *p* values summarized in Table 7.

### 5.1. Adult Zone

The first independent variable analyzed is “Arrive to Doctor (Adults),” which examines the time it takes for a patient in the adult zone of the ED to be seen by a doctor. Regression analysis focuses on key factors, such as triage bed availability, zone-specific nurse counts, and bed counts. The model integrates these factors to predict the arrival-to-doctor time for adult patients. As shown in Row 1 of Table 6, the model summary indicates a reasonably good fit with an *R*-squared value of 78.2. This suggests that the model explains approximately 80% of the variance in arrival-to-doctor times.

The ANOVA results, shown in the first two columns of Table 7, reveal significant effects for the number of zone-specific beds, indicating that this variable is a critical determinant of the arrival time to the doctor. In contrast, triage bed availability and zone-specific nurse counts, as well as their interactions, do not reach statistical significance, suggesting these variables may have less direct impact when considered independently. In addition, insights are drawn from the Pareto chart for the “Arrive to Doctor (Adults)” model. The chart highlighted beds as the most important factor in reducing the arrival-to-doctor times, followed by nurses.

**Figure 6:**
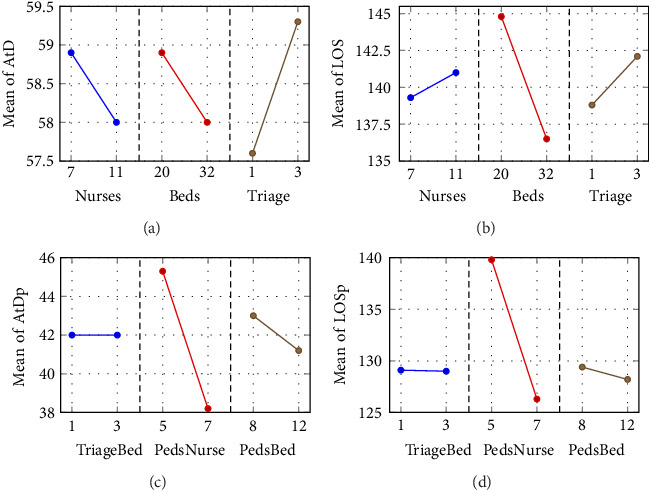
Main effects plots showing the influence of key variables on different performance metrics in the emergency department (ED). (a) Arrival to doctor in the adult zone (AtD), (b) length of stay in adults (LOS), (c) arrival to doctor in pediatrics (AtDp), and (d) length of stay in pediatrics (LOSp). The plots illustrate the effects of triage beds, nurse staffing levels, and bed availability on patient flow and service efficiency across different ED zones.

The plot of main effects in Figure 6(a) shows that the number of nurses leads to the most significant decrease in mean arrival-to-doctor times, indicating that improving staffing levels could substantially reduce wait times. Furthermore, the interaction plot reveals that the effect of the zone nurses on arrival times becomes more pronounced when there are fewer zone beds available, indicating a potential bottleneck in bed availability during these scenarios. These findings have important managerial implications for the adult zone of the ED. First, increasing the number of beds can directly reduce wait times. However, it is also essential to ensure adequate nurse staffing for these additional beds, as the interaction between nurse availability and bed capacity significantly affects patient flow. Hospitals should consider targeted staffing increases during peak periods or in specific zones to optimize patient throughput and reduce arrival-to-doctor times.

Using regression models and factorial design analysis, the impact of triage beds, zone-specific nurses, and zone-specific beds on the LOS in the adult area of the ED is analyzed. The *R*-square values suggest a moderately good fit with the data, indicating that while the model explains a substantial portion of the variance in LOS, there may be potential overfitting or multicollinearity among predictors. ANOVA shown in the first two columns of Table 7 highlights the significant influence of nurses, demonstrating their critical role in determining LOS. However, the interactions between the triage beds, the nurses in the zone, and the beds in the zone did not show a statistically significant impact, indicating that the main effects of the variables were more predictive of the LOS outcomes than their interactions. Furthermore, the Pareto chart identifies the need for nurse staff as the key driver of reducing LOS, while increasing bed capacity contributes less. Hence, staffing optimization, especially during peak times, is the most effective approach to reduce patient stay duration.

**Figure 7:**
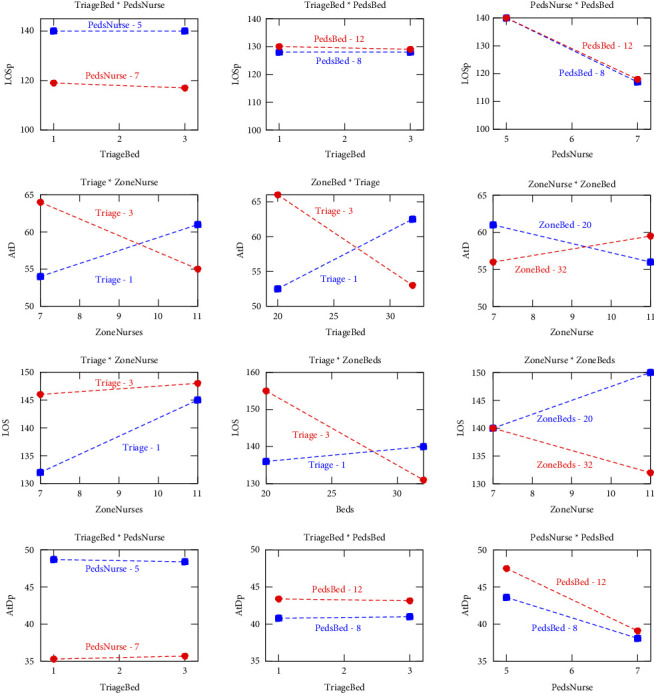
Interaction plots showing the impact of key variables on emergency department performance metrics. (a) Arrival to doctor in the adult zone (AtD), (b) length of stay in adults (LOS), (c) arrival to doctor in pediatrics (AtDp), and (d) length of stay in pediatrics (LOSp). Variables include triage beds, zone-specific nurses, and bed availability, highlighting their influence on patient flow and resource allocation.

In addition, the interaction plots (Figure 7(b)) provide deeper insights into the relationships between the variables. The main effect graph shows a marked decrease in LOS with an increase in the number of zone-specific nurses, underscoring the importance of nurse staffing levels in reducing LOS. Although the interaction plots indicate that the combination of factors influences LOS, individual factors, especially the number of nurses, have a more pronounced effect than their interactions.

### 5.2. Pediatric Zone

The analysis for “Arrive to Doctor (Peds)” in the pediatric zone of the ED used regression and factorial designs to assess the influence of triage beds, pediatric nurses (PedsNurse), and pediatric beds (PedsBed) on the time it takes for a pediatric patient to see a doctor. The model explains a significant part of the variance in arrival-to-doctor times, with an *R*-squared value of 84.8%, indicating a robust model fit and the effectiveness of the model in capturing the key dynamics that influence the flow of pediatric patients. The ANOVA results, shown in the last two columns of Table 7, highlight the significance of pediatric nurses and pediatric beds (PedsBed, *p* < 0.001), suggesting that these factors are crucial in determining arrival-to-doctor time for pediatric patients. However, interactions between these factors did not reach statistical significance (all *p* values > 0.05), indicating their combined effect does not add significant explanatory power beyond their individual contributions. Finally, the Pareto chart confirms that increasing pediatric nurse staffing is the most effective way to reduce wait times.

The plot of main effects (Figure 6(c)) further illustrates the importance of the availability of nursing staff, as the steep slope of PedsNurse indicates that increasing the number of pediatric nurses significantly decreases arrival-to-doctor times. Although the number of pediatric beds (PedsBed) also has a notable impact, the effect is less pronounced, suggesting that bed availability is a less sensitive factor compared to nurse staffing levels. The interaction plots show that the most significant decreases in wait times occur when both nursing staff and bed availability are optimized, although the statistical interaction between these factors remains nonsignificant.

Elements impacting the LOS for pediatric patients in the ED emphasize the role of triage beds, pediatric nurses (PedsNurse), and pediatric beds (PedsBed). Although no specific regression equation was provided, insights are drawn from ANOVA and other statistical outputs. The analysis reveals the significant influence of pediatric nurse availability on LOS, highlighting the crucial role of staffing in pediatric emergency care. ANOVA shown in the first two columns of Table 7 indicates that the model is significant (*F* value = 15.94, *p* value = 0.0), confirming the relevance of the variables chosen for the study. Specifically, the number of pediatric nurses (PedsNurse) shows a strongly significant effect (*p* value = 0.0) on LOS, while the number of triage beds and pediatric beds does not show a significant direct effect (*p* values > 0.05), nor do the interactions between these factors (all interaction *p* values > 0.05). Further insight from the Pareto chart confirms that pediatric nurses have the greatest impact on reducing LOS, with pediatric beds and triage beds contributing less significantly. This emphasizes that nurse staffing is the most influential factor in reducing LOS in pediatric care.

The plot of interaction plots (Figure 7(d)) illustrates the steep decline in LOS as the number of pediatric nurses increases, emphasizing that staffing is a key factor in reducing LOS in pediatric emergency care. They further show that although bed counts and triage bed availability also play roles, their impact is not as pronounced or statistically significant as that of nursing staff.

### 5.3. Testing and Implementation Phase: Strategic Recommendations for EDs

The testing and implementation phase leverages the results from the DOE to offer strategic recommendations to improve EDs. Across all zones, adult, pediatric, and triage, the analysis consistently showed that nurse staffing and bed availability are the key factors driving both LOS and arrival-to-doctor time. Nurse staffing, in particular, emerged as the most critical variable, especially in the pediatric zone, where increasing nurse availability significantly improves patient throughput. Although bed availability plays a role in reducing congestion, its impact is secondary to that of nurse staffing. Based on these findings, hospitals should prioritize optimizing nurse levels, particularly in the pediatric zone, to minimize patient wait times and improve overall operations. In the adult zone, the analysis emphasizes that while increasing bed numbers and adjusting the use of triage are beneficial, optimizing nurse staffing remains the most effective strategy to reduce LOS. Therefore, hospitals could adopt staffing models that allocate additional nurses to high-demand zones or peak times.

## 6. Discussion and Conclusions

### 6.1. Discussion

To build on these recommendations, hospital administrators may consider a comprehensive approach that integrates real-time adjustments in staffing and resource allocation based on patient volumes and acuity levels. A dynamic staffing model tailored to the specific needs of each zone, adult, pediatric, and triage could potentially lead to more efficient resource use, helping reduce both LOS and arrival-to-doctor time. Continuous monitoring of data and resource performance may be beneficial to ensure that adjustments remain effective over time. Further investigation of the interaction between nurse staffing and bed availability, along with factors such as patient severity and treatment protocols, may enhance the predictive power of the model and allow for more precise interventions. By following this adaptive approach, hospitals may improve how EDs operate in a way that ultimately improves patient care and satisfaction.

### 6.2. Implications for Theory and Practice

Practically, this study offers ED managers a systematic, evidence-based method to prioritize and implement changes. By identifying critical factors such as nurse staffing, resources can be strategically allocated to reduce wait times and improve throughput. Personalized interventions ensure alignment with the specific needs of different zones, while DT ensures patient-centered changes, enhancing both satisfaction and quality of care. Nonetheless, it is important for practitioners to evaluate the feasibility of certain interventions, such as increasing bed capacity, based on the specific context and constraints of their facility.

### 6.3. Limitations and Future Research Directions

This study has some limitations. First, the DES model only considered staffing levels and bed availability. Other factors, such as patient acuity, physician coverage, imaging service availability and timeliness, or external variables, may also influence the performance of the ED, but were not examined. Furthermore, the study did not account for external factors, such as variation in patient volumes or severity of conditions, which could affect the results. In addition, while patient satisfaction was mentioned in relation to wait time and LOS, detailed quantitative analysis of satisfaction scores was not included due to data limitations. This omission has been noted to maintain transparency and guide future research efforts.

In addition, the study focused on patients classified as ESI levels 3 and 4, as they represent the majority of ED visits. However, patients with ESI levels 1 and 2, although fewer in number, typically require more intensive resources and urgent interventions. The exclusion of these high-acuity patients limits the model's ability to capture the full complexity of ED operations. We have acknowledged this limitation and recommend expanding the scope in future modeling efforts.

Hence, future research should explore more comprehensive scenario generation methods, incorporating a wider range of factors to assess the resilience of ED under various conditions. Incorporating artificial intelligence and machine learning could further optimize resource allocation and performance by predicting patient needs. Additionally, future model iterations should integrate triage-based prioritization (e.g., ESI levels) to reflect urgency-based routing and resource competition, thereby improving clinical realism. Furthermore, replicating these findings in other healthcare settings and examining the long-term impacts on patient satisfaction will improve the generalizability of the framework. This research contributes to Industry 5.0 by integrating human-centered DT with advanced simulation and optimization techniques, promoting smarter and more resilient healthcare systems [39].

### 6.4. Concluding Remarks

In short, merging DT, DES, and DOE has proven to be a usefulstrategy to optimize the ED's performance. The study revealed that the number of nurses and beds significantly influences both the LOS and the arrival-to-doctor time, with the optimal combination of resources varying across different zones within the ED. While the current DES model focused primarily on staffing levels and bed availability, the integrated methodology provides a practical foundation for ED managers to make data-informed, patient-centered decisions. Using the principles of DT, DES, and DOE, EDs can better understand their operations and make informed choices to improve the quality of care they provide.

## Figures and Tables

**Table 1:** Key features and contributions of design thinking (DT), discrete event simulation (DES), and design of experiments (DOE).

Approach	Key features	Contribution in integration
DT	✓ Human-centered, iterative process	✓ Provides innovative, user-centric solutions
	✓ Empathy, ideation, and prototyping	✓ Identifies critical areas for improvement
	✓ Collaborative, cross-functional approach	✓ Identifies critical areas for improvement

DES	✓ Simulates individual events in complex systems	✓ Identifies bottlenecks and tests interventions
	✓ Detailed analysis of resources and patient flows	✓ Provides insights on operational efficiency and resource allocation
	✓ Captures variability and real-time processes	✓ Evaluates performance under stress (resilience testing)

DOE	✓ Systematic testing of factors and interactions	✓ Optimizes resource allocation (staff, beds)
	✓ Factorial design for multivariable analysis	✓ Assesses the impact of resource configurations to support data-driven decisions on the best interventions for ED improvement

**Table 2:** Time stamps sample from the regional hospital.

Arrive	Reg	Triage	Bed	Doc	Discharge	Room	Discharge status
01/04 0:03	01/04 0:18	01/04 0:11	01/04 0:07	01/04 0:19	01/04 3:01	Zone A	Discharged with approval
01/04 0:05	01/04 0:21	01/04 0:08	01/04 0:07	01/04 0:52	01/04 1:04	Zone A	Discharged with approval
01/04 0:07	01/04 0:14	01/04 0:14	01/04 0:12	01/04 0:27	01/04 2:37	Zone A	Discharged with approval
01/04 0:09	01/04 0:24	01/04 0:16	01/04 0:15	01/04 0:24	01/04 1:20	PEDS	Discharged with approval
01/04 0:12	01/04 0:26	01/04 0:24	01/04 0:15	01/04 0:23	01/04 2:00	PEDS	Discharged with approval

**Table 3:** Processing times and required resources for various tasks in the emergency department, including the associated staff and equipment needed for each task.

Task	Process time (min)	Staff/resource required						
		Physician	Zone bed	Zone nurse	Triage room	Triage nurse	Registration agent	Technician
Registration	TRI (2, 3, 5)						x	
Triage	1 + 2 ∗ Gamma (0.1, 2.5, 2.5)				x	x		
Nurse notes	1.7 ∗ TRIA (1, 0.3, 10.1)				x	x		
Physician consultation (Notes)	LogNORM (1.3, 2, 1)	x		x				
Physician consultation (Exam)	LogNORM (0.5, 1, 5.5)	x		x				
Nurse notes	Gamma (0.77, 0.4667, 13.24)			x				
Blood and radio test (CT)	TRIA (5, 10, 15)			x				x
Blood and radio test (Delay)	Gamma (0.77, 0.446, 14)			x				x
Blood and radio test (Xray)	TRIA (5, 7, 10)			x				x
Blood and radio test (Lab)	TRIA (5, 7, 10)			x				x
Reassessment (Doctor Exam)	LogNORM (0.5, 1,5. 5)	x	x	x				
Reassessment (Doctor Notes)	LogNORM (0.1, 1, 2)	x	x	x				
Reassessment (Nurse Notes)	Gamma (0.77, 0.4667, 13.24)	x	x	x				
Discharge process (Nurse Notes)	LogNORM (0.1, 1, 2)		x	x				
Discharge process (Discharge)	TRIA (10, 20, 30)		x	x				
Discharge process (Delay)	LogNORM (1, 2, 4.5)		x	x				

**Table 4:** Comparison of measured variables between 6 months of data and simulation results, showing the percentage differences in key performance metrics, including arrival-to-triage (AtT), arrival-to-doctor (AtD), and length of stay (LOS1 and LOS2).

Measured variable	Explanation	6 months	Simulations	Percentage difference (%)
AtT	Arrive to triage	15	15	0
AtD	Arrive to doctor	80	91	14
LOS1	Length of stay 1	184	193	4.8
LOS2	Length of stay 2	224	225	0

**Table 5:** Independent variables (DOE parameters) across different scenarios, comparing the current situation (Level 0), Scenario 1 (Level 1), and Scenario 2 (Level 2) with varying levels of nurse and bed resources in different zones of the emergency department.

Independent variables	Current situation (Level 0) (low level)	Scenario 1 (Level 1) (center point)	Scenario 2 (Level 2) (high level)
Number of nurses (adults)	7	9	11
Number of nurses (PEDS)	5	7	9
Number of beds (adults)	20	26	32
Number of beds (PEDS)	8	10	12
Number of beds (triage)	1	2	3

**Table 6:** Regression analysis results showing the regression equations and model summaries for the dependent variables: length of stay (LOS) for adults and pediatrics (PEDS), and arrival to doctor (AtD) for adults and pediatrics (AtD-P).

Dependent variable	Regression equation and model summary
LOS (adults)	LOS = −35.0 + 15.04 nurses + 4.68 beds + 52.6 triage
	−0.365 nurses × beds − 2.563 nurses × triage − 1.062 beds × triage
	*R*-sq (adj) = 78.22%

LOS (PEDS)	LOSp = 192 + 4.2 TriageBed − 9.8 Pedsnurse + 0.2 PedsBed
	−0.89 TriageBed × Pedsnurse − 0.26 TriageBed × PedsBed
	−0.09 Pedsnurse × PedsBed + 0.060 TriageBed × Pedsnurse × PedsBed
	*R*-sq = 85.45%

AtD (adults)	AtD = 10.71 − 0.375 nurses + 0.490 beds + 43.77 triage
	+0.1563 nurses × beds − 1.937 nurses × triage − 0.9792 beds × triage
	*R*-sq (adj) = 98.18%

AtD (PEDS)	AtDp = 86.5 + 1.8 TriageBed − 6.17 Pedsnurse − 2.56 PedsBed
	−0.42 TriageBed × Pedsnurse − 0.09 TriageBed × PedsBed
	+0.303 Pedsnurse × PedsBed + 0.026 TriageBed × Pedsnurse × PedsBed
	*R*-sq = 84.81%

*Note:* The *R*-squared values indicate the model's fit for each dependent variable.

**Table 7:** Analysis of variance (ANOVA) results for various performance metrics, including arrival-to-doctor (AtD), arrival-to-doctor for pediatrics (AtD-P), length of stay (LOS), and length of stay for pediatrics (LOS-P).

Equation	AtD		AtD-P		LOS		LOS-P	
Source	*F*	*p*	*F*	*p*	*F*	*p*	*F*	*p*
Model	6.41 × 10^1^	0.09 × 10^0^	1.52 × 10^1^	0 × 10^0^	0.59 × 10^1^	0.03 × 10^1^	5.19 × 10^1^	0 × 10^0^
Linear								
TriageBed	0.1 × 10^1^	0.05 × 10^1^	0 × 10^0^	9.55 × 10^1^	5.44 × 10^0^	0.25 × 10^0^	6 × 10^2^	8.14 × 10^1^
ZoneNurse	0.22 × 10^0^	0.072 × 10^1^	9.22 × 10^1^	0 × 10^0^	1 × 10^0^	0.05 × 10^1^	1.12 × 10^2^	0 × 10^0^
ZoneBed	0.54 × 10^1^	0.025 × 10^1^	1.10 × 10^1^	4 × 10^3^	1 × 10^0^	0.05 × 10^1^	7 × 10^2^	7.91 × 10^1^
2-way interactions								
TriageBed ∗ ZoneNurse	0.74 × 10^1^	0.022 × 10^1^	1.3 × 10^1^	7.26 × 10^1^	106 × 10^0^	0.06 × 10^0^	5 × 10^2^	8.25 × 10^1^
TriageBed ∗ ZoneBed	1.15 × 10^1^	0.018 × 10^1^	1.1 × 10^1^	7.45 × 10^1^	245 × 10^0^	0.04 × 10^0^	2 × 10^2^	8.86 × 10^1^
ZoneNurse ∗ ZoneBed	0.54 × 10^1^	0.025 × 10^1^	2.6 × 10^0^	1.23 × 10^1^	25 × 10^0^	0.012 × 10^1^	0 × 10^0^	9.69 × 10^1^

*Note:* The table includes *F* values and *p* values for linear, 2-way, and 3-way interactions of TriageBed, ZoneNurse, and ZoneBed.

## Data Availability

The data that support the findings of this study are available from the corresponding author upon reasonable request.
